# A commentary on the article entitled ‘Progress in the surgical treatment of sacrococcygeal pilonidal sinus’

**DOI:** 10.1097/JS9.0000000000001500

**Published:** 2024-05-03

**Authors:** Chu-Yun Xing, Ming-Yan Huang, Shi-Zhou Cheng

**Affiliations:** a Department of Pain Management, The First Affiliated Hospital of Yangtze University; b Department of Ophthalmology, The First Affiliated Hospital of Yangtze University, Jingzhou, Hubei Province, People’s Republic of China

*Dear Editor,*

We are interested in an article entitled ‘Progress in the surgical treatment of sacrococcygeal pilonidal sinus’ in the *International Journal of Surgery* ^1^. This paper used a systematic review and meta-analysis to include 27 articles, 54 studies, and 3612 participants for analysis after progress in literature screening. The aim of this study was to investigate issues related to the surgical treatment of sacrococcygeal pilonidal sinus (SPS), such as the selection of the optimal treatment plan for SPS, the necessity of abandoning the midline closure technique for the treatment of SPS, and the development of an individualized plan for patients. The authors conclude that there are several options for surgical treatment of SPS, including incisional drainage, resection of diseased tissue, and minimally invasive surgical procedures, but it is uncertain which surgical procedure should be considered the gold standard for treatment because even different researchers using the same surgical approach have produced conflicting results. However, it is certain that the incidence of postoperative recurrence and infection is much higher with the midline closure technique than with other techniques. Therefore, surgeons should develop an individualized plan that best suits the patient based on a comprehensive assessment of the patient’s wishes, the appearance of the SPS, and the surgeon’s professional ability. Nevertheless, in the meta-analysis methodology, there were several questions that we need to consider.

First, the authors searched only PubMed but not other important databases such as Web of Science, Embase, Cochrane Library, and Scopus. The flowchart of study selection provided by the authors is relatively simple, such that much important information about the progress of literature screening is not fully presented. Second, for most of the pooled outcomes, such as LF and KF techniques as well as modified and minimally invasive treatment techniques, the number of included studies was small, and the sample size was small. In addition, most meta-analysis results are negative, even though it is appropriate to perform a meta-analysis at this time. Third, due to the fewer possible pooled outcomes, most of the outcomes in the included studies are presented with a summary table. However, we did not directly see a significant *P* value in every reported outcome among these included studies^2^. Finally, even in the absence of publication bias, it is possible to bias the results due to the relatively small number of studies pooled^3^.

Overall, the authors did not conduct a more systematic search strategy. In this meta-analysis^1^, the authors did not perform a quality assessment for evidence. Because evidence deriving from randomized trials typically initially starts with a provisional grade of high strength of evidence (SOE), whereas evidence deriving from observational studies initially starts with a provisional grade of low SOE^4^. Whereas the authors conducted a meta-analysis by including both randomized trials and observational studies, it is difficult to assess the SOE.

## Ethical approval

Not applicable.

## Consent

Not applicable.

## Sources of funding

Not applicable.

## Author contribution

C.-Y.X. and M.-Y.H.: equally participated in the original idea and writing the first draft; S.-Z.C.: edited the final manuscript. All authors approved the last version of this manuscript.

## Conflicts of interest disclosure

There are no conflicts of interest among the authors.

## Research registration unique identifying number(UIN)

Not applicable.

## Guarantor

Shi-Zhou Cheng.

## Data availability statement

Data sharing is not applicable to this article as no new data were created or analyzed in this study.

## Provenance and peer review

Commentary, internally reviewed.
